# Frequency-Weighted EIS Manifold Learning for Lithium-Ion Battery Remaining Useful Life Prediction

**DOI:** 10.3390/s26196206

**Published:** 2026-09-30

**Authors:** Tianze Wang, Ying Zhang, Hanyue Du, Huan Wang, Wenxian Yang

**Affiliations:** 1 School of Mechanical Engineering, University of Science and Technology Beijing, Beijing 100083, China; u202440577@xs.ustb.edu.cn; 2 School of Information Science and Engineering, Shandong University, Qingdao 266237, China; duhanyue@mail.sdu.edu.cn; 3 Department of Industrial Engineering, Tsinghua University, Beijing 100084, China; hwan36@cityu.edu.hk; 4 Centre for Efficiency and Performance Engineering, School of Computing and Engineering, University of Huddersfield, Huddersfield HD1 3DH, UK; w.yang@hud.ac.uk

**Keywords:** lithium-ion battery, remaining useful life prediction, electrochemical impedance spectroscopy, kernel principal component analysis, nonlinear EIS representation

## Abstract

Accurate remaining useful life (RUL) prediction of lithium-ion batteries is essential for ensuring the reliability and safety of battery management systems. However, conventional electrical and thermal signals are sensitive to operating conditions, while linear feature extraction methods may not adequately characterize the nonlinear electrochemical aging information embedded in electrochemical impedance spectroscopy (EIS). Moreover, the unequal degradation relevance of different impedance-frequency regions is rarely considered. To address these limitations, this paper proposes a frequency-weighted EIS manifold-learning framework for battery RUL prediction. A band-level frequency-weighting strategy is first introduced to incorporate degradation-related frequency priors, after which kernel principal component analysis (KPCA) is employed to construct compact nonlinear representations of EIS evolution, followed by support vector regression (SVR) with hyperparameter optimization. Linear bias correction and moving-average smoothing are further incorporated to improve prediction consistency. Experiments on seven batteries within the investigated LR2032 dataset, including one chronological test and six held-out-cell tests, demonstrate that the proposed framework consistently outperforms the baseline models, achieving an average MSE of 59.434 cycles^2^ and an average $R^{2}$ of 0.989.

## 1. Introduction

Accurate prediction of lithium-ion battery remaining useful life (RUL) is a central task in battery health management because reliable degradation assessment supports safe operation and advanced battery management strategies [1,2,3,4,5].

Battery degradation is inherently nonlinear and influenced by multiple interacting factors, including charge/discharge rates, temperature, and usage conditions, which collectively shape complex aging behaviors that may be difficult to characterize using simple linear models [6,7]. Existing RUL estimation approaches can be broadly classified into physics-based and data-driven methods [8,9]. Physics-based approaches construct electrochemical or equivalent-circuit models (ECMs) to represent internal aging mechanisms [10,11,12]. Although these models enable mechanistic interpretation and can support long-term predictions, their performance typically hinges on accurate parameter identification, strong model assumptions, and computationally intensive simulations, which can limit their real-time deployment under variable operating conditions [13]. By contrast, data-driven methods rely solely on operational data and leverage statistical learning, machine learning, or deep learning algorithms to extract degradation-relevant features and infer RUL [14,15,16,17]. For example, Severson et al. [18] achieved high-accuracy cycle-life prediction using early-cycle signatures, while Roman et al. [19] developed BHUMP, a feature-engineering pipeline using raw charge-curve segments for health estimation. More recent work has also explored transfer-learning-based battery health estimation to improve model adaptability across operating conditions [20]. Data-driven models offer greater flexibility and avoid the need for complex physical modeling [21]. However, most data-driven battery prognostics rely on conventional electrical or thermal signals (i.e., current, voltage, and temperature). These signals exhibit notable limitations: current measurements can be sensitive to load fluctuations; voltage responses depend on instantaneous SOC and discharge rate rather than degradation alone; and temperature measurements may exhibit delayed responses to internal aging processes and can be influenced by environmental conditions [22]. Although these signals provide useful information for battery monitoring, their susceptibility to external disturbances and limited feature stability can constrain their effectiveness for long-term RUL prediction under complex degradation patterns [23]. Electrochemical impedance spectroscopy (EIS) has been increasingly investigated as a diagnostic technique for probing electrode kinetics, ion diffusion, charge-transfer resistance, and solid–electrolyte interphase (SEI) evolution across a wide frequency spectrum [24,25]. By measuring battery impedance at various frequencies, EIS can provide information about electrochemical processes, material degradation, and ion transport characteristics, thereby supporting battery-state assessment. For instance, Xiaojia Su et al. [26] applied the step-wave method to identify low-frequency EIS measurements online, extracting health indicators related to the lithium-ion diffusion coefficient. Compared with conventional time-domain measurements, EIS provides a frequency-resolved characterization of battery responses by applying small perturbations over a broad frequency range. Although time-domain electrical signals also contain degradation-related information, they are strongly coupled with operating conditions such as load variation, state of charge, and temperature. In contrast, EIS analyzes impedance responses at different frequencies, enabling the characterization of electrochemical processes associated with ohmic resistance, charge transfer, and ion diffusion. Therefore, EIS should be regarded as a complementary diagnostic perspective that provides frequency-dependent information for battery prognostics rather than a replacement for conventional measurements [27,28]. Despite its advantages, EIS measurement data are high-dimensional and may exhibit nonlinear evolution across aging cycles [29,30], which can complicate their application in battery diagnostics. In such high-dimensional data, extracting the most relevant information while eliminating redundancy is crucial for improving model accuracy [31].

Existing dimensionality reduction methods, such as principal component analysis (PCA), can reduce redundancy in high-dimensional data but rely on linear assumptions [32]. When applied to battery datasets that exhibit nonlinear degradation behaviors [33], PCA may not adequately represent the curved and frequency-dependent evolution of impedance features, which may lead to limited degradation representations. Handling high-dimensional, nonlinear, and partially redundant data therefore remains an important challenge for battery health prediction. In the context of EIS, this challenge becomes even more pronounced. EIS spectra span a wide frequency range, and their temporal evolution can form manifold-like trajectories in the Nyquist space as the battery ages. These trajectories exhibit nonlinear transformations, such as arc expansion, curvature changes, and low-frequency diffusion-tail growth, that may be difficult to represent using a single linear projection. Kernel principal component analysis (KPCA) maps the original spectra into a high-dimensional feature space through kernel functions [34], providing a nonlinear dimensionality-reduction mechanism for representing such structural variations. In this study, KPCA is therefore used as a nonlinear feature representation method rather than being assumed to preserve any specific manifold geometry by construction. Different from studies that use KPCA primarily to compress predefined battery health indicators, the present work applies KPCA to frequency-weighted EIS spectra so that frequency relevance is incorporated before nonlinear representation.

In addition, although EIS provides electrochemical information across a wide frequency spectrum, not all frequency regions contribute equally to battery degradation characterization [35,36,37]. In a conventional qualitative interpretation, the high-frequency region is dominated mainly by ohmic and other fast-response contributions, the mid-frequency arc is strongly associated with interfacial and charge-transfer behavior, and the low-frequency region reflects slower transport and diffusion processes. Because these electrochemical processes overlap in frequency, the band boundaries used in this study should be understood as operational partitions rather than strict mechanistic boundaries. The more pronounced aging-related changes observed in the mid- and low-frequency portions motivate incorporating frequency-region relevance before nonlinear representation learning [38]. Existing EIS-based RUL studies have explored different strategies for extracting degradation information from impedance spectra. Li et al. [39] developed an EIS-based deep learning approach to learn degradation-related representations directly from impedance measurements, whereas Yuan et al. [23] transformed EIS information into a distribution-of-relaxation-times representation and constructed degradation indicators for subsequent RUL prediction. Related EIS-based studies have addressed frequency relevance through feature selection, partial-spectrum analysis, or regression-based relevance estimation. In particular, Zhang et al. [40] employed automatic relevance determination (ARD) within a Gaussian-process framework to evaluate the relevance of individual impedance-frequency features. In contrast, the present study uses explicit band-level weighting before KPCA, so that frequency relevance affects the nonlinear EIS representation before the subsequent RUL regression. Therefore, the proposed framework separates frequency-region relevance modeling, nonlinear representation, and RUL prediction into successive modeling stages rather than embedding frequency relevance solely within the final regression model.

Overall, existing EIS-based RUL methods often overlook the nonlinear evolution of impedance spectra and the unequal degradation relevance of different frequency regions. To address these limitations, this study proposes an EIS-specific framework that integrates band-level frequency weighting with nonlinear representation and RUL regression. The main contributions are summarized as follows:

(1) A frequency-aware nonlinear EIS representation framework is developed where band-level weighting introduces degradation-related frequency priors before KPCA-based feature extraction.
(2) Frequency weighting is coupled with KPCA-based nonlinear representation so that frequency-region relevance is incorporated before constructing compact degradation features from the high-dimensional EIS spectra.
(3) The integrated KPCA–SVR framework is validated on seven batteries, demonstrating consistent performance in chronological and held-out-cell prediction.

The remainder of this paper is organized as follows. Section 2 introduces the fundamental principles of EIS and its relevance to battery degradation analysis. Section 3 presents the proposed frequency-weighted nonlinear manifold learning framework for RUL prediction. Section 4 presents the experimental evaluation and comparative analysis of the proposed method. Finally, Section 5 summarizes the main findings and concludes the paper.

## 2. EIS for Battery Degradation Analysis

This section introduces the electrochemical basis of EIS and analyzes the degradation-related characteristics of different frequency regions. These observations provide the physical motivation for the frequency-aware weighting strategy used in the proposed framework.

### 2.1. Fundamentals of Electrochemical Impedance Spectroscopy

EIS characterizes the dynamic response of a battery by applying a small-amplitude sinusoidal excitation current (or voltage) over a wide range of frequencies and measuring the resulting voltage (or current) response [41]. The resulting impedance spectrum, typically spanning from the millihertz to megahertz range, reflects multiple electrochemical processes occurring at different time constants [42]. Under steady-state sinusoidal excitation, the voltage and current can be written as $u\left(t\right)=U_{0}\sin\left(\omega t+\varphi_{u}\right)$ and $i\left(t\right)=I_{0}\sin\left(\omega t+\varphi_{i}\right)$, respectively. The complex impedance is defined by the ratio of their phasors rather than by the pointwise ratio of the two instantaneous waveforms:

(1) $$Z\left(j\omega \right)=\frac{\underline{U}\left(j\omega \right)}{\underline{I}\left(j\omega \right)}=\frac{U_{0}}{I_{0}}\exp\;\left[j\left(\varphi_{u}-\varphi_{i}\right)\right]=\left|Z\left(\omega \right)\right|\exp\left(j\varphi_{Z}\right),$$

where $\underline{U}\left(j\omega \right)$ and $\underline{I}\left(j\omega \right)$ are the voltage and current phasors, $U_{0}$ and $I_{0}$ are their amplitudes, ω is the angular frequency, and $\varphi_{Z}=\varphi_{u}-\varphi_{i}$ is the voltage–current phase difference. For a fixed excitation frequency, Equation (1) is independent of time. The impedance can equivalently be decomposed into its real and imaginary components [43]:

(2) $$Z\left(j\omega \right)=Z^{\prime }\left(\omega \right)+jZ^{\prime \prime }\left(\omega \right)=\left|Z\left(\omega \right)\right|\left[\cos\varphi_{Z}+j\sin\varphi_{Z}\right],$$

where $Z^{\prime }$ represents the real component and $Z^{\prime \prime }$ represents the imaginary component of the impedance. A Nyquist plot conventionally displays $Z^{\prime }$ against $-Z^{\prime \prime }$. The high-frequency intercept corresponds primarily to ohmic resistance, the mid-frequency arc reflects interfacial and charge-transfer processes, and the low-frequency tail is associated mainly with mass-transport and diffusion behavior.

As the battery ages, the morphology of the Nyquist curve evolves nonlinearly: semicircular arcs gradually expand due to increasing charge-transfer resistance, while the low-frequency tail becomes more pronounced as ion diffusion slows and the SEI layer thickens. These progressive trajectory shifts across cycles contain information associated with electrochemical degradation. Consequently, the sequence of Nyquist curves can be viewed as a high-dimensional, manifold-like trajectory associated with battery aging.

These characteristics motivate the use of feature extraction techniques that can characterize nonlinear structural variations in the impedance spectrum [44]. Traditional linear dimensionality reduction methods may be limited in representing the curved, frequency-dependent evolution of EIS data. In contrast, nonlinear approaches can provide greater flexibility for representing these degradation trajectories, potentially providing more informative features for subsequent RUL prediction.

### 2.2. Frequency-Domain Feature Analysis of EIS Spectra

**Figure 1:**
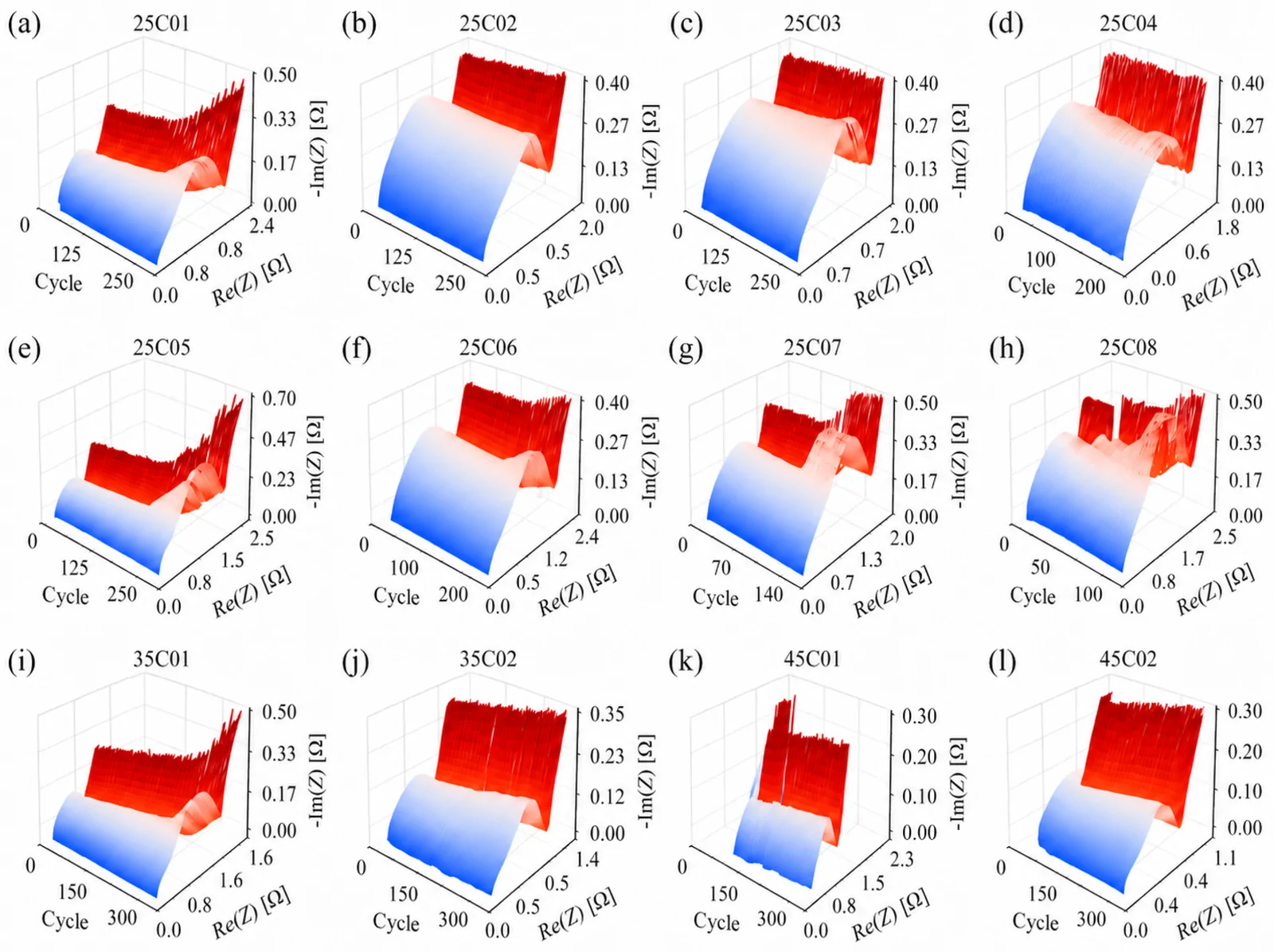
Evolution of EIS spectra over aging cycles for twelve cells: (**a**) 25C01, (**b**) 25C02, (**c**) 25C03, (**d**) 25C04, (**e**) 25C05, (**f**) 25C06, (**g**) 25C07, (**h**) 25C08, (**i**) 35C01, (**j**) 35C02, (**k**) 45C01, and (**l**) 45C02. Cell identifiers combine the ambient temperature and cell index; for example, 25C01 denotes cell 01 aged at 25 °C. The three axes denote cycle index, real impedance Re(Z) (Ω), and negative imaginary impedance −Im(Z) (Ω), respectively.

Figure 1 illustrates the cycle-dependent evolution of the EIS spectra for all twelve cells. As seen in Figure 1a–c, the impedance surfaces of 25C01–25C03 change progressively with the cycle index, and the variations are concentrated around the curved and rising portions of the trajectories rather than appearing as a uniform translation of the entire spectrum. Similar cycle-dependent evolution can be observed in Figure 1d–h for the remaining 25 °C cells and in Figure 1i–l for the 35 °C and 45 °C cells. At the same time, the magnitude and shape of the trajectories differ among individual cells, indicating both a common degradation tendency and cell-dependent variability.

From an electrochemical perspective, the high-, mid-, and low-frequency regions of an EIS spectrum are mainly associated with ohmic resistance, charge-transfer behavior, and ion-diffusion processes, respectively [45]. The relatively limited changes near the high-frequency intercept suggest that this region alone may provide insufficient degradation discrimination. In contrast, the more visible expansion and curvature changes in the arc and tail portions are consistent with the evolution of charge-transfer resistance, SEI-related processes, and diffusion behavior during aging [46]. Therefore, the observations in Figure 1 suggest that the EIS spectra contain degradation-relevant information with nonlinear and frequency-dependent characteristics.

Based on these visual observations, treating all frequency components equally may underemphasize the spectral regions that exhibit larger aging-related changes. This motivates the frequency-aware weighting strategy adopted in this study, in which greater emphasis is assigned to the low- and mid-frequency components before nonlinear feature extraction [39].

## 3. Methodology

**Figure 2:**
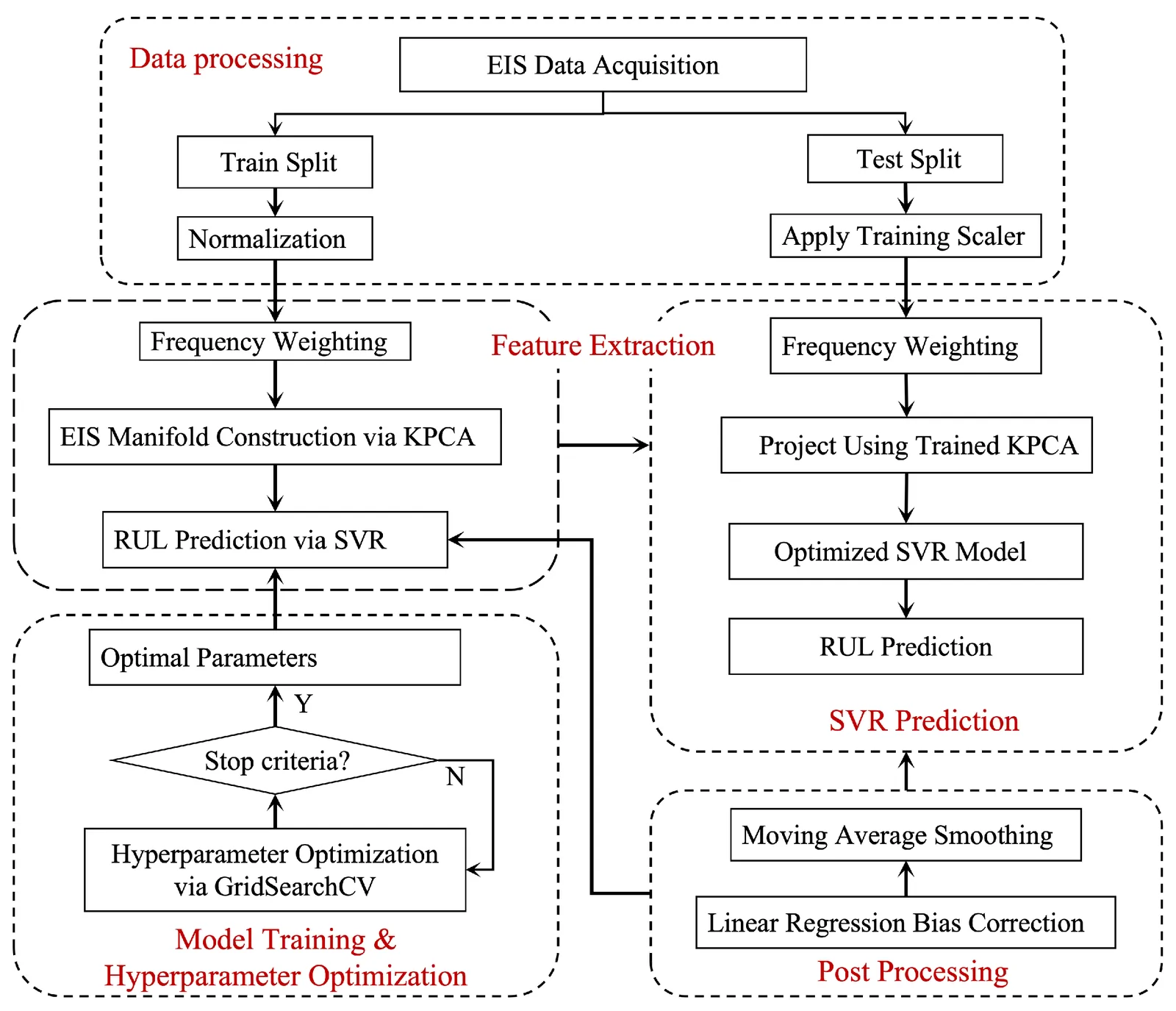
Overall workflow of the proposed frequency-weighted EIS manifold-learning framework. All data-dependent transformations are fitted using model-development data and then reused for validation and test samples; the test branch does not refit the normalization or KPCA models.

This section presents the proposed frequency-weighted nonlinear EIS representation and RUL prediction framework. As illustrated in Figure 2, the method first incorporates frequency-region relevance through band-level weighting, then extracts nonlinear degradation representations using KPCA, and finally performs SVR-based RUL regression followed by bias correction and moving-average smoothing. To avoid fragmented presentation, the method is organized into three functional parts: frequency-weighted nonlinear representation, SVR-based regression and parameter selection, and prediction post-processing.

### 3.1. Frequency-Weighted Nonlinear EIS Representation

Let $x_{n}={\left[x_{n}\left(f_{1}\right),x_{n}\left(f_{2}\right),\ldots ,x_{n}\left(f_{d}\right)\right]}^{⊤}\in R^{d}$ denote the preprocessed EIS feature vector of the *n*-th sample, where *d* is the number of frequency-dependent features, $f_{i}$ is the *i*-th measurement frequency, and $x_{n}\left(f_{i}\right)$ is the corresponding impedance-derived feature. Because different frequency regions exhibit different aging sensitivities, a weighted vector is constructed as

(3) $$x_{n}^{w}=Wx_{n},$$

where

(4) $$W=\mathrm{diag}\;\left(w\left(f_{1}\right),w\left(f_{2}\right),\ldots ,w\left(f_{d}\right)\right)$$

is a diagonal weighting matrix. The frequency-dependent weight is defined by

(5) $$w\left(f_{i}\right)=\left\{\begin{array}{cc} w_{\mathrm{low}}, & f_{i}<100\;\mathrm{Hz},\\ w_{\mathrm{mid}}, & 100\;\mathrm{Hz}\leq f_{i}<10\;\mathrm{kHz},\\ w_{\mathrm{high}}, & f_{i}\geq 10\;\mathrm{kHz}, \end{array}\right.$$

where $w_{\mathrm{low}}$, $w_{\mathrm{mid}}$, and $w_{\mathrm{high}}$ are dimensionless weights assigned to the low-, mid-, and high-frequency regions, respectively. The 100 Hz and 10 kHz cutoffs were selected as coarse operational boundaries based on the frequency-dependent characteristics of the measured EIS spectra and the conventional interpretation of different spectral regions. In the measured frequency range of 0.02 Hz–20 kHz, the low-frequency region below 100 Hz mainly captures the diffusion- and transport-related impedance evolution, while the intermediate-frequency region reflects pronounced interfacial and charge-transfer-related responses. The frequencies above 10 kHz mainly correspond to high-frequency ohmic and fast-response contributions, which exhibit relatively limited aging-related variation in the present dataset. The 100 Hz and 10 kHz cutoffs are operational band boundaries adopted for the present dataset; they are not intended to imply a strict one-to-one separation of electrochemical mechanisms. In this study, $w_{\mathrm{low}}>w_{\mathrm{mid}}>w_{\mathrm{high}}$ is adopted based on the observed spectral evolution and validation results: aging-related variations are more pronounced in the low- and mid-frequency regions than in the high-frequency region, while the low-frequency weight exhibits the largest influence on validation performance within the tested weighting grid. This ordering is therefore used as a dataset-specific weighting strategy rather than a universal rule for EIS-based battery prognosis.

Given *N* weighted training samples $\left\{x_{1}^{w},x_{2}^{w},\ldots ,x_{N}^{w}\right\}$, KPCA maps each sample into a reproducing kernel Hilbert space F through a nonlinear mapping $\phi :R^{d}\to F$. The kernel function is

(6) $$\kappa \left(x_{i}^{w},x_{j}^{w}\right)={\langle \phi \left(x_{i}^{w}\right),\phi \left(x_{j}^{w}\right)\rangle }_{F},$$

and the kernel matrix $K\in R^{N\times N}$ is defined by $K_{ij}=\kappa \left(x_{i}^{w},x_{j}^{w}\right)$. The centered kernel matrix is

(7) $$K_{c}=HKH,\;H=I_{N}-\frac{1}{N}11^{⊤},$$

where $I_{N}$ is the N×N identity matrix, and 1 is an *N*-dimensional vector of ones.

The *r*-th KPCA direction is obtained from

(8) $$K_{c}u^{\left(r\right)}=\lambda_{r}u^{\left(r\right)},\;{\left(u^{\left(r\right)}\right)}^{⊤}u^{\left(r\right)}=1,$$

where $\lambda_{r}>0$ and $u^{\left(r\right)}$ are the corresponding eigenvalue and Euclidean-normalized eigenvector, respectively.

For a new weighted sample $x^{w}$, define its raw kernel vector with respect to the *N* training samples as

(9) $$k\left(x^{w}\right)={\left[\begin{array}{ccc} \kappa (x_{1}^{w},x^{w}) & \cdots & \kappa (x_{N}^{w},x^{w}) \end{array}\right]}^{⊤}.$$

Because the eigendecomposition is performed on the centered training kernel matrix, the new-sample kernel vector must be centered using only the training-kernel statistics:

(10) $$\begin{array}{cc} \tilde{k}\left(x^{w}\right)= & k\left(x^{w}\right)-\frac{1}{N}K1-\frac{1}{N}11^{⊤}k\left(x^{w}\right)\\ \; & +\frac{1}{N^{2}}11^{⊤}K1. \end{array}$$

The *r*-th KPCA score of the new sample is then

(11) $$z_{r}\left(x^{w}\right)=\frac{{\left(u^{\left(r\right)}\right)}^{⊤}\tilde{k}\left(x^{w}\right)}{\sqrt{\lambda_{r}}}.$$

Here, the factor $1/\sqrt{\lambda_{r}}$ is consistent with the Euclidean normalization in Equation (8). All kernel means in Equation (10) are estimated from the training set and are subsequently reused without refitting for the validation and test samples. The retained *q* scores form the nonlinear feature vector $z={\left[z_{1},z_{2},\ldots ,z_{q}\right]}^{⊤}\in R^{q}$.

An RBF kernel is adopted:

(12) $$\kappa \left(x_{i}^{w},x_{j}^{w}\right)=\exp\left(-\frac{∥x_{i}^{w}-x_{j}^{w}{∥}_{2}^{2}}{2\sigma_{\mathrm{KPCA}}^{2}}\right),$$

where $\sigma_{\mathrm{KPCA}}>0$ is the KPCA kernel bandwidth.The RBF kernel is selected because the multi-cycle EIS samples exhibit a nonlinear and smoothly evolving degradation structure that cannot be fully represented by a linear projection. By measuring similarity through the Euclidean distance between frequency-weighted EIS samples, the RBF kernel enables KPCA to capture curved degradation trajectories without imposing a predefined global functional form on the nonlinear mapping. Moreover, the kernel bandwidth $\sigma_{\mathrm{KPCA}}$ controls the locality of the similarity measure and is selected through cross-validation using only the model-development data.

**Figure 3:**
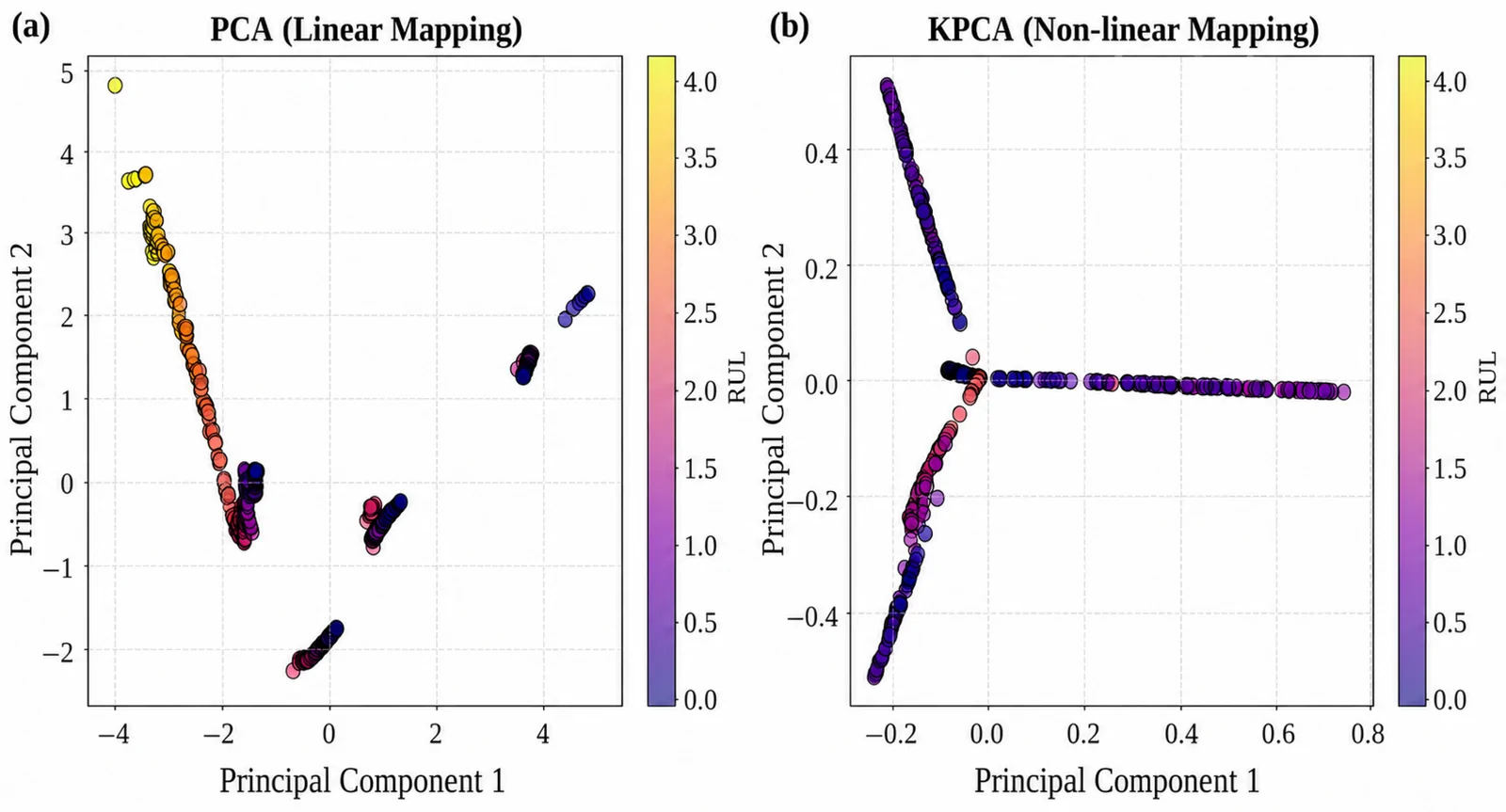
Two-dimensional degradation-feature projections obtained using (**a**) PCA and (**b**) KPCA. The color scale denotes the normalized RUL.

Figure 3 compares the two-dimensional representations obtained using PCA and KPCA, with color indicating the normalized RUL. In Figure 3a, the PCA features form several separated curved clusters, and samples with different RUL values remain locally mixed in parts of the projection. This indicates that a single linear projection does not fully unfold the nonlinear evolution of the EIS samples. By contrast, Figure 3b shows that the KPCA features are reorganized into several branch-like trajectories, along which the RUL colors change more continuously. The comparison therefore suggests that the nonlinear mapping makes the degradation-related ordering of the samples more explicit in the projected space. For visualization, the RUL values in Figure 2 are normalized to the range [0,1] by subtracting the minimum RUL value and dividing by the difference between the maximum and minimum RUL values.

More specifically, the gradual variation of the RUL color along the KPCA trajectories indicates that neighboring samples in the learned feature space generally correspond to similar degradation stages, while progression along a trajectory is accompanied by a systematic change in RUL. Because the RUL labels are not used in constructing the KPCA representation, this ordered color progression provides qualitative evidence that the nonlinear EIS features retain degradation-related information inherent in the impedance spectra. Such an RUL-related organization of the feature space provides a structured representation for the subsequent SVR model to learn the mapping from KPCA features to RUL. This interpretation is qualitative, since the two-dimensional projection does not imply a strictly monotonic relationship between any individual KPCA component and RUL.

### 3.2. SVR-Based RUL Regression and Parameter Selection

Let $z_{i}\in R^{q}$ denote the KPCA feature vector of the *i*-th training sample, and let $y_{i}$ denote its RUL label. SVR estimates the nonlinear mapping from $z_{i}$ to $y_{i}$ using the ϵ-insensitive loss

(13) $$L_{\epsilon }\left({\hat{y}}_{i},y_{i}\right)=\left\{\begin{array}{cc} 0, & |{\hat{y}}_{i}-y_{i}|\leq \epsilon ,\\ |{\hat{y}}_{i}-y_{i}|-\epsilon , & |{\hat{y}}_{i}-y_{i}|>\epsilon , \end{array}\right.$$

where ${\hat{y}}_{i}$ is the predicted RUL, and ϵ≥0 defines the width of the insensitive tube.

In the SVR feature space, the regression function is written as

(14) $$f\left(z\right)={\langle w_{\mathrm{SVR}},\psi \left(z\right)\rangle }_{H_{\mathrm{SVR}}}+b,$$

where ψ(·) maps the KPCA feature vector into the reproducing kernel Hilbert space $H_{\mathrm{SVR}}$, $w_{\mathrm{SVR}}\in H_{\mathrm{SVR}}$ is the regression weight, ${\langle \cdot ,\cdot \rangle }_{H_{\mathrm{SVR}}}$ denotes the inner product in that space, and b∈R is the bias term. The complete primal optimization problem is

(15) $$\begin{array}{cc} \min_{w_{\mathrm{SVR}},b,\xi ,\xi^{*}}\; & \frac{1}{2}{∥w_{\mathrm{SVR}}∥}_{H_{\mathrm{SVR}}}^{2}+C\sum_{i=1}^{N}\left(\xi_{i}+\xi_{i}^{*}\right)\\ s.t.\;\; & y_{i}-f\left(z_{i}\right)\leq \epsilon +\xi_{i},\\ \; & f\left(z_{i}\right)-y_{i}\leq \epsilon +\xi_{i}^{*},\\ \; & \xi_{i}\geq 0,\;\xi_{i}^{*}\geq 0,\;i=1,\ldots ,N. \end{array}$$

where C>0 controls the trade-off between model flatness and errors outside the ϵ-insensitive tube. The slack variable $\xi_{i}$ measures an underprediction beyond the tube, whereas $\xi_{i}^{*}$ measures an overprediction beyond the tube.

By introducing the dual variables $\eta_{i}$ and $\eta_{i}^{*}$, the resulting predictor is

(16) $$\hat{y}\left(z\right)=\sum_{i=1}^{N}\left(\eta_{i}-\eta_{i}^{*}\right)\kappa_{\mathrm{SVR}}\left(z_{i},z\right)+b,$$

where $0\leq \eta_{i},\eta_{i}^{*}\leq C$, $\sum_{i=1}^{N}\left(\eta_{i}-\eta_{i}^{*}\right)=0$, and $\kappa_{\mathrm{SVR}}$ is the SVR kernel. The symbols $\eta_{i}$ and $\eta_{i}^{*}$ are used here to avoid confusion with the KPCA eigenvectors. For an RBF SVR kernel,

(17) $$\kappa_{\mathrm{SVR}}\left(z_{i},z_{j}\right)=\exp\left(-\gamma_{\mathrm{SVR}}{∥z_{i}-z_{j}∥}_{2}^{2}\right),$$

where $\gamma_{\mathrm{SVR}}>0$ controls the kernel width.

The SVR hyperparameters $\theta =(C,\epsilon ,\gamma_{\mathrm{SVR}})$ are selected through GridSearchCV. For a candidate configuration θ, the mean *M*-fold cross-validation score is

(18) $$S_{\mathrm{CV}}\left(\theta \right)=\frac{1}{M}\sum_{m=1}^{M}S_{m}\left(\theta \right),$$

where $S_{m}\left(\theta \right)$ is the validation score on the *m*-th fold. The selected configuration is

(19) $$\theta^{*}=\arg\max_{\theta \in \Theta }S_{\mathrm{CV}}\left(\theta \right),$$

where Θ is the predefined parameter grid. The model is then retrained on the complete training set using $\theta^{*}$.

### 3.3. Prediction Correction, Smoothing, and Integrated Workflow

Let ${\hat{y}}_{t}^{\mathrm{SVR}}$ denote the SVR prediction for the *t*-th sample. A linear correction model is used to adjust systematic prediction offset. The correction parameters are estimated using only the training and validation predictions as

(20) $$\left(a^{*},c^{*}\right)=\arg\min_{a,c}\frac{1}{N_{c}}\sum_{t=1}^{N_{c}}{\left[y_{t}-\left(a{\hat{y}}_{t}^{\mathrm{SVR}}+c\right)\right]}^{2},$$

where $N_{c}$ is the number of samples used to estimate the correction parameters. The corrected prediction is then defined as

(21) $${\hat{y}}_{t}^{\mathrm{corr}}=a^{*}{\hat{y}}_{t}^{\mathrm{SVR}}+c^{*}.$$

No ground-truth RUL label from the test set is used to estimate $a^{*}$ or $c^{*}$.

A causal moving-average filter is subsequently applied. For samples indexed by t=1,2,…, let

(22) $$L_{t}=\min\left(L,t\right),\;{\tilde{y}}_{t}=\frac{1}{L_{t}}\sum_{j=0}^{L_{t}-1}{\hat{y}}_{t-j}^{\mathrm{corr}},$$

where *L* is the predefined maximum smoothing-window length, $L_{t}$ is the number of corrected predictions available at sample *t*, and ${\tilde{y}}_{t}$ is the final smoothed RUL estimate. Thus, the filter uses only the current and preceding predictions and does not access future test samples.

By integrating frequency-aware weighting with nonlinear KPCA representation, the proposed framework addresses two modeling issues: the unequal degradation relevance of different EIS frequency regions and the nonlinear evolution of impedance trajectories. The subsequent SVR, linear correction, and moving-average modules are used to model the feature–RUL relationship, adjust systematic offsets, and reduce short-term fluctuations, respectively. Their quantitative effects and the overall predictive performance are evaluated in Section 4.

## 4. Experimental Validation

This section evaluates the proposed framework on seven battery cells from the investigated LR2032 dataset through one within-cell chronological prediction task and six completely held-out-cell tests. It describes the dataset and experimental protocol, analyzes the frequency-weighting parameters, compares the complete framework with four baseline models, and examines its predictive performance across cells aged at 25, 35, and 45 °C. A component-wise and progressive ablation study is further conducted on three representative cells (25C02, 35C02, and 25C07), followed by repeated cross-validation stability analysis and visualization of the learned EIS degradation representations.

### 4.1. Data Description and Experimental Protocol

The proposed method was evaluated using the open-access battery-aging dataset provided by Zhang et al. [40]. The dataset contains EIS and cycle-aging measurements from twelve 45-mAh Eunicell LR2032 lithium-ion coin cells with a LiCoO_2_/graphite chemistry. The cells were aged at three ambient temperatures: 25 °C (25C01–25C08), 35 °C (35C01–35C02), and 45 °C (45C01–45C02).

Each aging cycle followed a constant-current/constant-voltage (CC–CV) charging protocol at 1C (45 mA) to 4.2 V, followed by constant-current discharging at 2C (90 mA) to 3.0 V. EIS spectra were recorded every even-numbered cycle using a 5-mA excitation current over the frequency range from 0.02 Hz to 20 kHz. The source dataset contains measurements at nine predefined charging and discharging states. Following Zhang et al. [40], only State V is used in this study. State V corresponds to the fully charged condition after a 15-min open-circuit rest; spectra from the other eight states are not combined with the State V samples.

For cell *b*, the end-of-life (EOL) cycle is defined as the first cycle at which the measured discharge capacity falls below 80% of its initial reference capacity:

(23) $$c_{\mathrm{EOL}}^{\left(b\right)}=\min\left\{c:Q^{\left(b\right)}\left(c\right)<0.8Q_{0}^{\left(b\right)}\right\},$$

where $Q^{\left(b\right)}\left(c\right)$ is the measured discharge capacity at cycle *c*, and $Q_{0}^{\left(b\right)}$ is the initial reference capacity of cell *b*. The RUL label assigned to an EIS spectrum recorded at physical cycle $c_{i}$ is

(24) $$y_{i}^{\left(b\right)}={\mathrm{RUL}}^{\left(b\right)}\left(c_{i}\right)=\max\left(c_{\mathrm{EOL}}^{\left(b\right)}-c_{i},0\right).$$

Thus, RUL is expressed in remaining charge–discharge cycles rather than in the number of remaining EIS measurements or as a normalized quantity. Each EIS spectrum is aligned with its RUL label through its recorded physical cycle number; the chronological sample index is used only for plotting and is not used to calculate the RUL label.

The measurement and labeling conventions generally follow Zhang et al. [40], whereas the present study adopts a modified model-development and evaluation protocol. Cells 25C01, 25C03, 25C04, 35C01, and 45C01, together with the early-cycle State V samples of 25C02, are used for model development. The remaining later-cycle samples of 25C02 form a chronological test set for evaluating future-cycle prediction within the same cell. For battery 25C02, the retained EIS sequence contained 525 samples and was chronologically divided at 70% of the sequence. The first 367 samples were used for model development, while the remaining 158 samples were reserved for chronological testing.

Cells 25C05, 25C06, 25C07, 25C08, 35C02, and 45C02 are completely excluded from model fitting, frequency-weight selection, SVR hyperparameter optimization, linear bias correction, and all other data-dependent parameter-selection procedures. All retained State V samples from these six cells are used only for held-out-cell evaluation. Therefore, the complete evaluation contains one within-cell chronological test on 25C02 and six held-out-cell tests spanning the three ambient temperatures of 25, 35, and 45 °C. This protocol is designed to assess both future-cycle prediction within a partially observed cell and transfer to previously unseen cells under different aging conditions.

### 4.2. Model Optimization and Implementation Details

To select the frequency-weighting ratio and SVR parameter configuration within the predefined search ranges, a joint search method was employed because the selected SVR parameters may change with different weighting ratios, and conversely, the selected weighting ratio may vary with different SVR parameters. The early-cycle training and validation samples of battery 25C02 were used for the frequency-weight sensitivity analysis. The later-cycle samples of 25C02 were not involved in parameter selection and were reserved for chronological testing. Cells 25C05–25C08, 35C02, and 45C02 were kept completely unseen during frequency-weight selection and model optimization and were used only for held-out-cell evaluation. The EIS spectrum was divided into three frequency bands: low frequency (below 100 Hz), mid frequency (100 Hz to 10 kHz), and high frequency (above 10 kHz). In a series of controlled experiments, the weight of the high-frequency band was fixed at 1, while the weights of the low- and mid-frequency bands were systematically varied from 1 to 10. The coefficient of determination ($R^{2}$) was used to quantify the proportion of variance in the true RUL values explained by the predictions.

**Figure 4:**
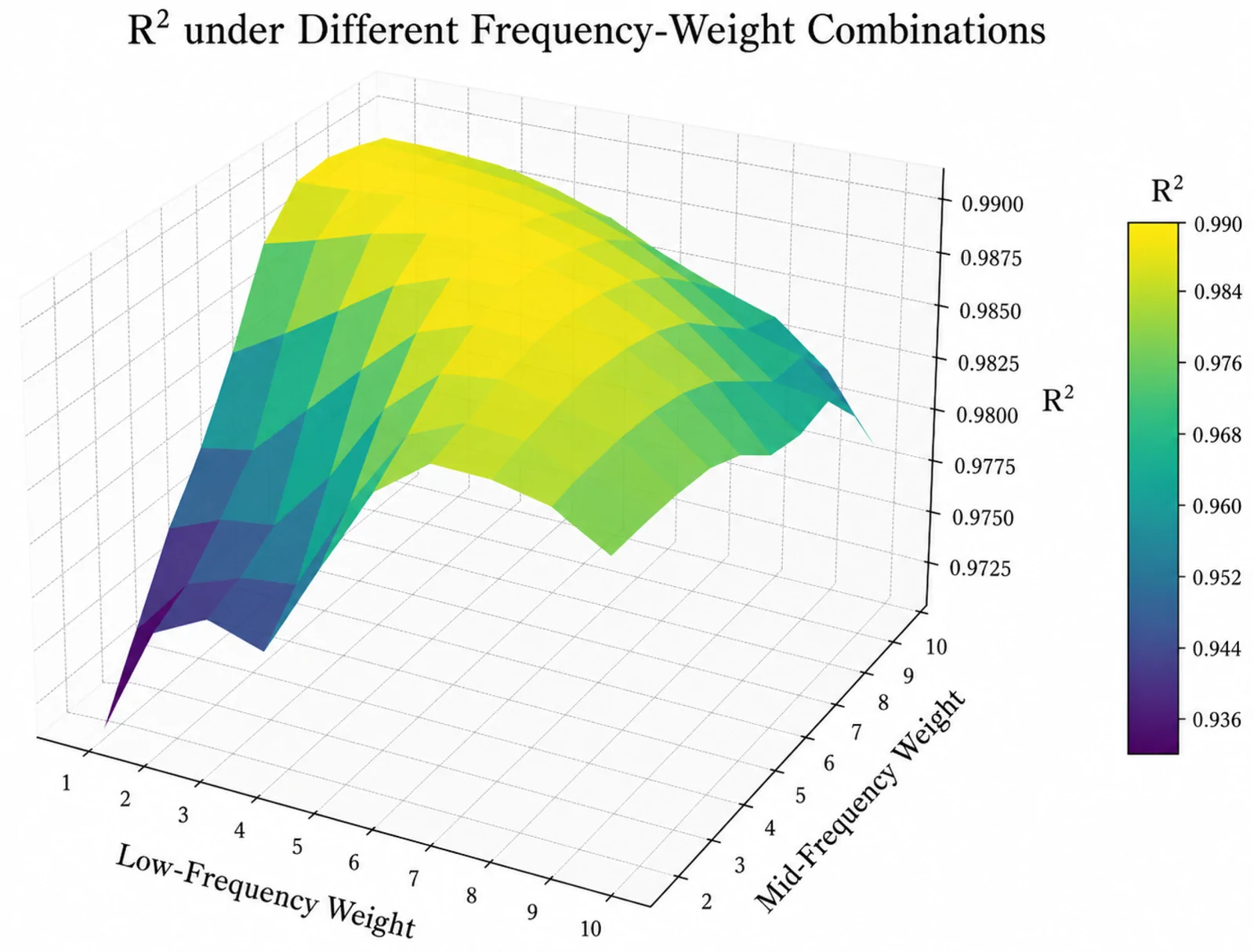
Effect of the dimensionless low- and mid-frequency weights on the validation $R^{2}$ score; the high-frequency weight is fixed at 1.

Figure 4 shows the validation $R^{2}$ values obtained for different combinations of low- and mid-frequency weights, while the high-frequency weight is fixed at 1. The yellow region in the performance surface corresponds to higher $R^{2}$ values, whereas the blue–purple region corresponds to lower values. When the low-frequency weight increases from 1 to a moderate level, the surface rises rapidly, particularly when the mid-frequency weight is also greater than 1. After the low-frequency weight becomes excessively large, the surface tends to flatten or decrease slightly. In comparison, varying the mid-frequency weight produces a smaller change in the surface height over most of the tested range.

These observations indicate that the low-frequency components have the largest influence on the validation performance within the tested grid, while the mid-frequency components provide a secondary contribution. The highest validation $R^{2}$ is obtained near the combination $\left(w_{\mathrm{low}},w_{\mathrm{mid}},w_{\mathrm{high}}\right)=\left(6,5,1\right)$. This ratio was selected using only the early-cycle training and validation samples of 25C02 and was subsequently fixed for the later-cycle 25C02 test and for all six held-out-cell evaluations. Therefore, the ratio 6:5:1 is reported as the best-performing configuration under the adopted 25C02 validation protocol rather than as a universal optimum for every battery or operating temperature.

To further examine whether the identified frequency-priority trend depends on a specific battery trajectory, an additional post hoc consistency analysis was conducted using two completely held-out cells, 35C02 and 45C02. This analysis was performed only to evaluate the consistency of the frequency-weighting trend across different aging conditions and was not involved in frequency-weight selection or model optimization. Following the original weighting procedure, the high-frequency weight was fixed at 1, and the relative contributions of low- and mid-frequency regions were independently examined for each cell.

As summarized in Table 1, the representative weighting trends observed for 35C02 and 45C02 were approximately 6:4:1 and 7:5:1, respectively, compared with the selected 6:5:1 configuration from 25C02. Although the exact ratios varied slightly among different cells, all three batteries consistently exhibited the same frequency-priority trend, where the low-frequency region received the highest importance, followed by the mid-frequency region and the high-frequency region.

Before model training, all EIS features were standardized using statistics estimated exclusively from the model-development data. The frequency weights for the low-, mid-, and high-frequency regions were fixed at a ratio of 6:5:1. Both KPCA and SVR employed RBF kernels, and their principal hyperparameters were selected through five-fold cross-validation over predefined search ranges. The configuration achieving the highest mean validation $R^{2}$ was adopted for the final evaluation.

The linear bias-correction parameters were estimated without using any test labels, after which a causal moving-average filter was applied to the corrected predictions. All baseline models were optimized independently under the same data-partition and evaluation protocol, and all methods were implemented under a unified computational framework to ensure consistency in data processing, model optimization, and performance evaluation.

All experiments were implemented in Python 3.13.5 under Windows 11. The main software packages included NumPy 2.1.3, SciPy 1.15.3, and scikit-learn 1.6.1. The experiments were conducted on a computer equipped with an AMD Ryzen 9 7945HX processor (2.50 GHz), 16 GB of RAM, and an NVIDIA GeForce RTX 4060 Laptop GPU with 8 GB of video memory. The proposed frequency-weighted KPCA–SVR framework was executed entirely on the CPU without GPU acceleration.

The retained KPCA dimensionality was set to q=10. For the RBF kernel in KPCA, the kernel parameter γ was searched over $\left\{10^{-3},10^{-2},10^{-1},1,10\right\}$. For the RBF-SVR model, the hyperparameters were optimized using GridSearchCV over C∈{1,10,100,1000}, ϵ∈{0.01,0.05,0.1,0.2}, and $\gamma \in \{10^{-3},10^{-2},10^{-1},1\}$. The selected hyperparameters were C=100, ϵ=0.1, and γ=0.01. The moving-average window length was set to L=5. For the baseline models, the ANN consisted of two fully connected hidden layers with 64 and 32 neurons, respectively, using ReLU activation. The LSTM model employed a single LSTM layer with 64 hidden units followed by a fully connected output layer. For GPR, an RBF kernel was adopted, and its kernel hyperparameters were optimized during model fitting.

The main computational cost of the proposed framework arises from the kernel-based representation and regression stages. For *N* training samples with *d*-dimensional EIS features, construction of the RBF kernel matrix for KPCA requires approximately $O(N^{2}d)$ operations and $O(N^{2})$ memory. A full eigendecomposition of the kernel matrix requires up to $O(N^{3})$ operations, although the practical cost is reduced when only a limited number of principal components are retained. The computational cost of SVR training is data-dependent and is typically between quadratic and cubic with respect to the number of training samples. In contrast, the frequency-weighting, linear bias-correction, and moving-average stages introduce only minor additional computational overhead.

Under the above hardware environment, the complete hyperparameter optimization, including five-fold cross-validation, required approximately 8.4 s, while fitting the final model using the selected parameters required approximately 0.6 s. After offline training, the average inference time was approximately 0.5 ms per EIS sample. These results indicate that the main computational burden is concentrated in the offline model-development stage, whereas the online prediction cost remains modest for the investigated dataset.

### 4.3. Performance Evaluation and Model Comparison

Five methods were compared for lithium-ion battery RUL prediction: support vector regression (SVR), Gaussian process regression (GPR), long short-term memory (LSTM) networks, artificial neural networks (ANN), and the proposed frequency-weighted KPCA–SVR framework. In this comparison, SVR denotes the conventional direct-regression baseline using raw EIS features, whereas proposed denotes the complete framework including frequency weighting, KPCA representation, RBF–SVR regression, linear bias correction, and moving-average smoothing. All methods were trained and evaluated under the same data-partition protocol using mean absolute error (MAE), mean squared error (MSE), and the coefficient of determination ($R^{2}$). Each model underwent individual hyperparameter tuning using only the model-development data.

**Figure 5:**
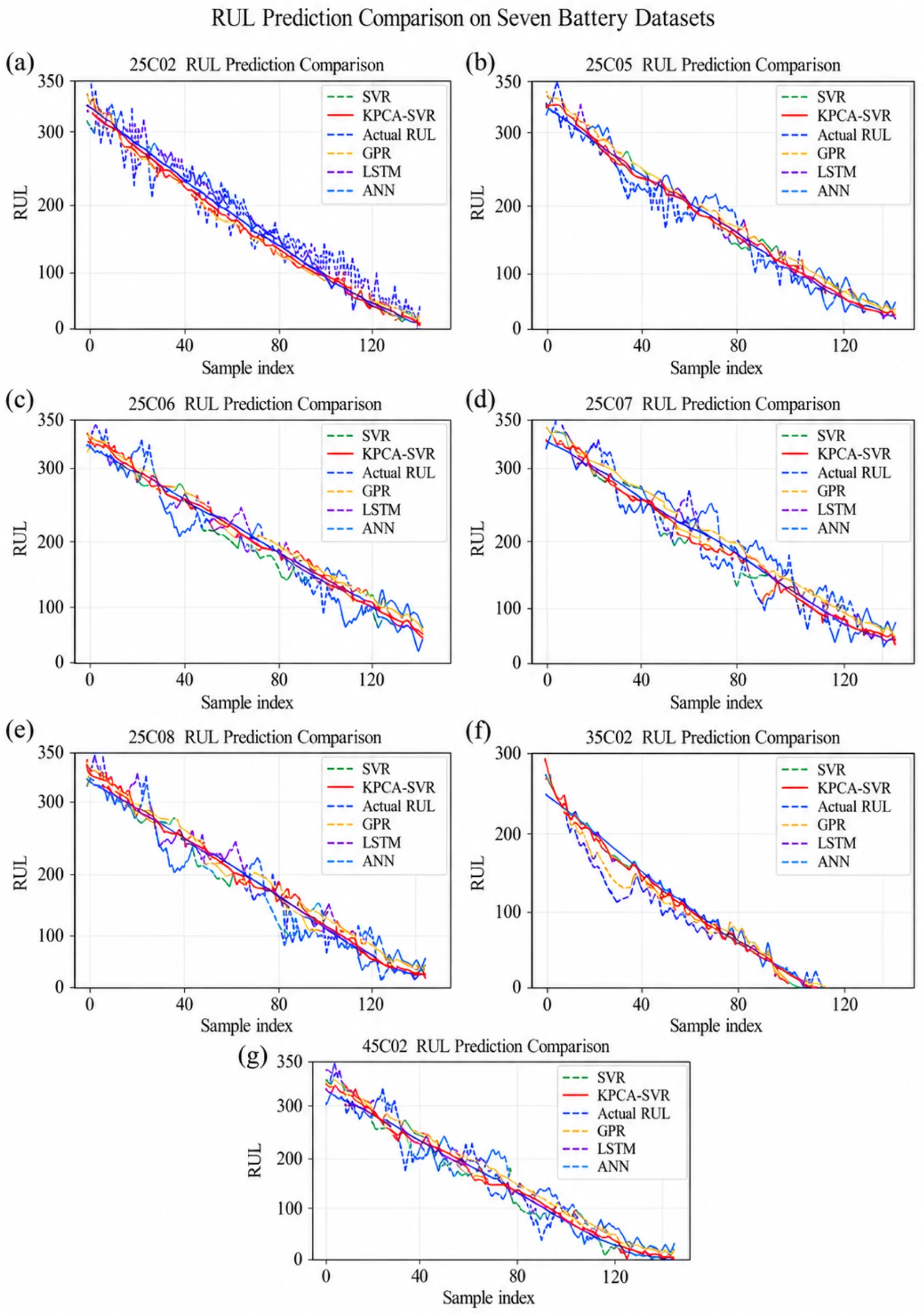
RUL prediction comparisons on seven battery datasets: (**a**) 25C02, (**b**) 25C05, (**c**) 25C06, (**d**) 25C07, (**e**) 25C08, (**f**) 35C02, and (**g**) 45C02. The horizontal axis denotes the chronological index of the retained State V EIS samples, while the vertical axis represents the remaining useful life (RUL) in charge–discharge cycles. Battery 25C02 is evaluated using a within-cell chronological test, whereas the other six batteries are evaluated as completely held-out cells.

The corresponding RUL prediction curves for the seven evaluation batteries are presented in Figure 5. RUL is expressed in remaining charge–discharge cycles, whereas the horizontal axis denotes the chronological index of the retained State V EIS samples. The 25C02 result represents within-cell chronological prediction using its later-cycle samples, while 25C05–25C08, 35C02, and 45C02 represent predictions on completely held-out cells.

The prediction curves show that all five methods generally capture the decreasing RUL tendency, but their local accuracy and trajectory stability differ substantially. ANN and LSTM exhibit relatively large oscillations or systematic deviations on several batteries. GPR and the direct SVR baseline provide more stable predictions but still show visible errors in parts of the middle- and late-life regions. In comparison, the proposed method follows the actual RUL trajectory more closely across the majority of the sample indices and maintains comparatively stable predictions near the end-of-life region.

Table 2 presents the quantitative comparison across the seven evaluation batteries. The proposed framework achieves the lowest MAE and MSE and the highest $R^{2}$ on every battery, indicating that its performance advantage is consistent across the evaluated cells and temperatures within the investigated dataset.

For reproducibility, the model configurations, major hyperparameters, and training settings used in the comparative experiments are summarized in Table 3.

Across all seven datasets, the proposed method obtains MAE values ranging from 4.240 to 7.747 cycles, MSE values ranging from 35.098 to 88.828 cycles^2^, and $R^{2}$ values ranging from 0.984 to 0.994. The corresponding mean MAE, MSE, and $R^{2}$ are 6.239 cycles, 59.434 cycles^2^, and 0.989, respectively. Thus, the method maintains a relatively narrow performance range despite cell-to-cell and temperature-dependent differences in the EIS degradation trajectories.

On the chronological test samples of 25C02, the proposed method achieves an MAE of 6.216 cycles, an MSE of 51.625 cycles^2^, and an $R^{2}$ of 0.990. GPR is the strongest baseline on this cell, with an MAE of 8.113 cycles and an MSE of 84.572 cycles^2^. Relative to GPR, the proposed method reduces MAE by 23.4% and MSE by 39.0%.

For the six completely held-out cells, the proposed method achieves an average MAE of 6.243 cycles, an average MSE of 60.735 cycles^2^, and an average $R^{2}$ of 0.989. Relative to the strongest baseline selected separately for each battery, the MAE reductions range from 28.9% to 33.4%, while the MSE reductions range from 44.3% to 52.2%. The lowest held-out-cell error is obtained on 35C02, with an MAE of 4.240 cycles and an MSE of 35.098 cycles^2^, whereas the lowest held-out-cell $R^{2}$ remains 0.984 on 25C07.

Overall, compared with the strongest baseline on each of the seven batteries, the proposed framework reduces MAE by 23.4%–33.4% and MSE by 39.0%–52.2%. These results demonstrate that the improvements are maintained not only for within-cell chronological prediction but also across previously unseen cells aged at different ambient temperatures within the evaluated dataset.

Zhang et al. [40] evaluated an EIS-based GPR model with automatic relevance determination (ARD) using a different experimental protocol, including 25C01–25C04 as training cells and 25C05–25C08 as testing cells for the 25 °C evaluation. Their ARD formulation provides feature-level relevance values for individual impedance frequencies, whereas the present method introduces explicit band-level weights for the low-, mid-, and high-frequency regions before nonlinear KPCA representation. Because the train–test splits, regression models, and uncertainty formulations differ, the numerical results are not directly comparable. This paper therefore does not claim an accuracy or computational advantage over the GPR/ARD model without a same-protocol reproduction.

Although the preceding comparison demonstrates the overall performance of the complete framework, it does not by itself identify which components are responsible for the observed gains. Therefore, the following subsection examines the effects of frequency weighting, PCA or KPCA representation, linear bias correction, and moving-average smoothing through controlled ablation configurations.

### 4.4. Component-Wise and Progressive Ablation Study

Because conducting the complete progressive ablation on all seven batteries would substantially increase the number of experimental configurations, three representative cells were selected for component analysis. Battery 25C02 represents within-cell chronological prediction, 35C02 represents a completely held-out cell at 35 °C, and 25C07 provides an additional held-out 25 °C case with comparatively larger prediction errors. Together, these cases allow the component effects to be examined under both chronological and cross-cell evaluation settings.

Raw EIS with a tuned RBF–SVR model was used as the reference configuration. PCA and KPCA were first evaluated without frequency weighting to compare linear and nonlinear feature extraction. Frequency weighting was then evaluated alone and in combination with PCA or KPCA. Finally, linear bias correction and moving-average smoothing were progressively added to the weighted KPCA–RBF–SVR configuration. The same training, validation, and test partitions were maintained for all configurations. The last two rows in Table 4 are cumulative additions rather than independent stand-alone modules.

The comparison between PCA and KPCA shows that nonlinear feature extraction provides more consistent benefits than linear dimensionality reduction. On 25C02, PCA increases the MAE and MSE of the raw-EIS baseline from 9.490 and 122.207 to 10.126 and 143.684, respectively, whereas KPCA reduces them to 7.731 and 81.956. On 35C02, both PCA and KPCA improve upon the raw-EIS baseline, with KPCA obtaining the lower MAE and MSE of 5.143 and 55.908. On 25C07, PCA again degrades the raw-EIS baseline, whereas KPCA reduces MAE from 12.472 to 10.411 and MSE from 233.060 to 169.760. These results indicate that the nonlinear KPCA representation is more robust across the three representative cases than the linear PCA projection.

Frequency weighting alone does not produce a uniform improvement. It reduces the MAE and MSE of the raw-EIS model on 25C02 but increases both errors on 35C02 and 25C07. The weighted-PCA configuration also shows mixed behavior across the three cells. Therefore, the weighting operation should not be interpreted as an independently effective transformation under every cell condition.

A more consistent improvement is observed when frequency weighting is combined with KPCA. Relative to the unweighted KPCA–RBF–SVR configuration, the weighted KPCA configuration reduces MAE from 7.731 to 6.784 and MSE from 81.956 to 61.903 on 25C02; from 5.143 to 4.572 and from 55.908 to 43.776 on 35C02; and from 10.411 to 8.612 and from 169.760 to 113.270 on 25C07. The corresponding $R^{2}$ values increase from 0.984 to 0.988, from 0.990 to 0.992, and from 0.969 to 0.980, respectively. This consistent pattern across all three representative cells supports a complementary relationship between degradation-sensitive frequency emphasis and nonlinear KPCA representation.

Adding linear bias correction further reduces MAE and MSE on all three cells. Relative to weighted KPCA–RBF–SVR, MAE decreases from 6.784 to 6.083 on 25C02, from 4.572 to 4.118 on 35C02, and from 8.612 to 7.548 on 25C07. The correction-only cumulative configuration therefore yields the lowest MAE on all three representative batteries, indicating that a small systematic offset remains in the uncorrected regression output.

After moving-average smoothing is applied, MSE is further reduced to 51.625, 35.098, and 88.828, while $R^{2}$ increases to 0.990, 0.994, and 0.984 on 25C02, 35C02, and 25C07, respectively. MAE increases slightly relative to the correction-only configuration on all three cells. This pattern indicates a trade-off: causal smoothing suppresses relatively large local deviations and improves squared-error-based metrics, while the associated temporal lag can introduce small errors over a larger number of samples.

Compared with the raw-EIS RBF–SVR reference configuration, the complete framework reduces MAE and MSE by 34.5% and 57.8% on 25C02, by 28.9% and 51.7% on 35C02, and by 37.9% and 61.9% on 25C07. Overall, the three-cell ablation results identify KPCA as the most consistently beneficial representation component and demonstrate that the contribution of frequency weighting is mainly realized through its interaction with nonlinear KPCA representation. The frequency weighting provides degradation-related spectral priors, while KPCA transforms these weighted spectra into compact nonlinear degradation representations. These component-level observations explain the construction of the complete framework, while the seven-battery comparison provides broader evidence for its predictive performance.

### 4.5. Stability and Representation Analysis

To further examine the stability of the proposed KPCA–SVR model with respect to data partitioning, five-fold cross-validation was repeated seven times, yielding 35 fold-level validation $R^{2}$ scores. This analysis is used as an internal stability assessment of the model-development procedure and is not regarded as independent evidence of cross-cell generalization, which is evaluated separately using the completely held-out cells.

**Figure 6:**
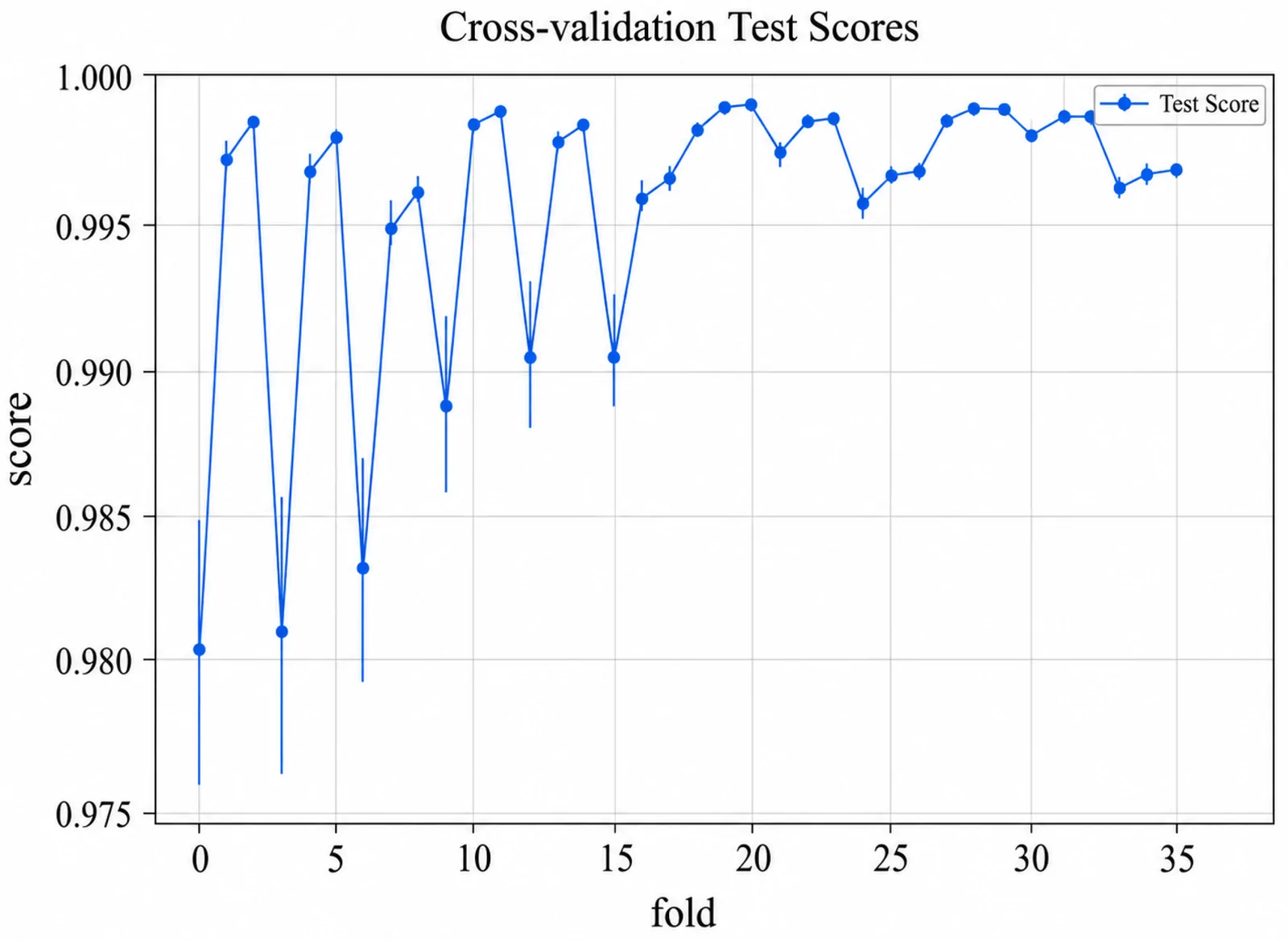
Distribution of 35 fold-level $R^{2}$ scores obtained from five-fold cross-validation repeated seven times. The horizontal axis denotes the fold index, and the vertical axis denotes the dimensionless validation $R^{2}$ score.

Figure 6 shows that most fold-level $R^{2}$ scores are concentrated near the upper end of the range, while only a small number of folds exhibit comparatively lower values. The overall concentration of the scores indicates limited variation across the majority of the repeated partitions, although several lower-scoring folds show that the model remains sensitive to the specific data split. Therefore, this experiment is interpreted as supplementary evidence of internal model stability rather than as a substitute for the held-out-cell evaluation.

In addition to the repeated cross-validation analysis, the learned low-dimensional feature space was examined to assess whether the KPCA representation preserves an ordered degradation progression across cells.

**Figure 7:**
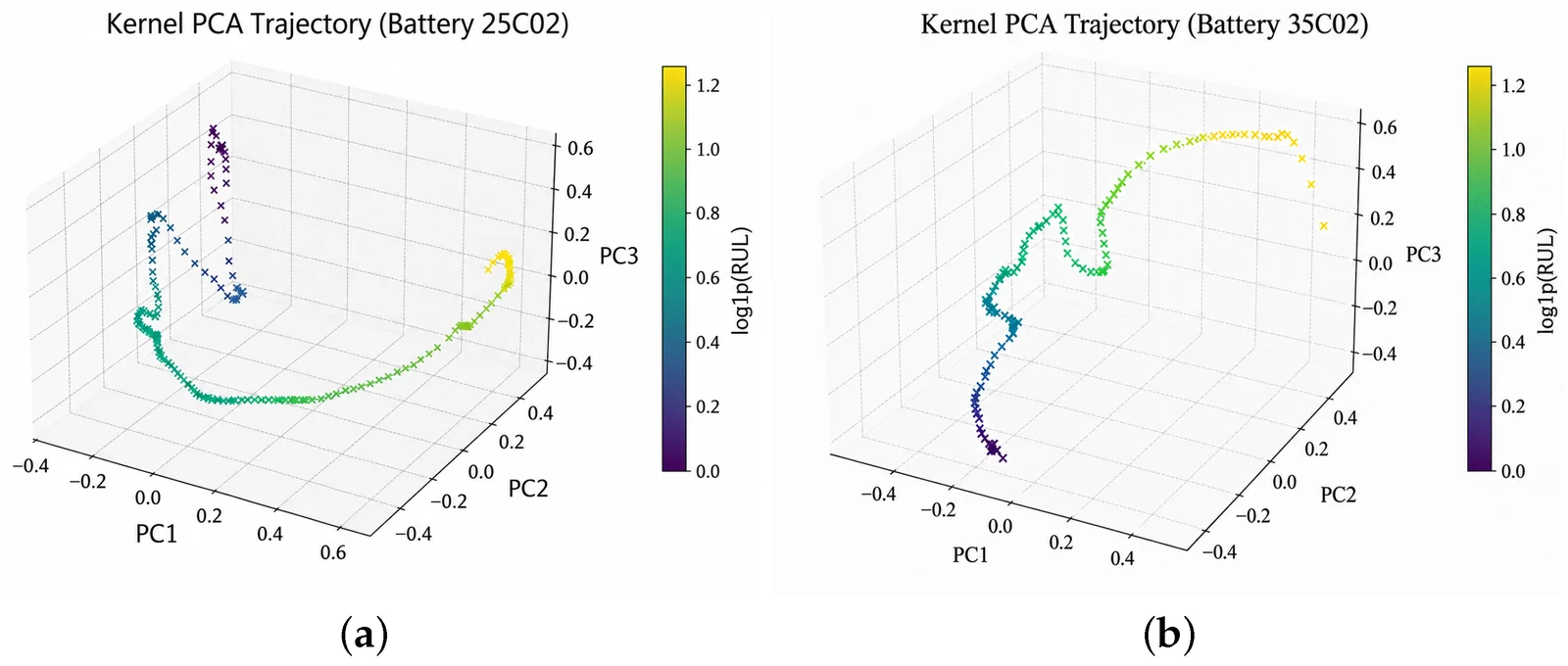
Three-dimensional EIS manifold trajectories for (**a**) 25C02 and (**b**) 35C02. The axes denote the first three KPCA components, and the color scale denotes the log1p-normalized RUL.

To qualitatively examine the learned low-dimensional representations, the extracted features were visualized in a three-dimensional KPCA space, as shown in Figure 7. In Figure 7a, the 25C02 samples form a continuous curved trajectory, and the color changes gradually along the path with degradation progression. Figure 7b shows a different geometric trajectory for the held-out 35C02 cell, but a similarly ordered color progression is maintained. For visualization, the RUL values in are first min–max normalized to the range [0,1] by subtracting the minimum RUL value and dividing by the difference between the maximum and minimum RUL values. The normalized values are then transformed using log(1+x) for color-scale visualization.

The two panels therefore exhibit cell-dependent manifold shapes together with a common RUL-related ordering. The absence of large isolated jumps suggests that nearby points in the learned space generally correspond to nearby degradation stages. This observation provides qualitative support for using the KPCA features as degradation representations in the subsequent SVR model, while the different shapes in Figure 7a,b also indicate that cross-cell variability is preserved rather than artificially removed.

### 4.6. Discussion and Limitations

The results indicate that frequency-aware weighting and nonlinear KPCA representation can provide complementary benefits for EIS-based RUL prediction, particularly when evaluated on completely held-out cells. Nevertheless, the scope of the present validation should be interpreted carefully. First, all cells originate from a single LR2032 LiCoO_2_/graphite coin-cell dataset, so the present results do not establish transferability to other chemistries, cell formats, or manufacturing batches. Second, only State V EIS spectra measured under a controlled fully charged condition after a 15-min open-circuit rest are used; performance under arbitrary SOC, dynamic load, or online measurement conditions remains to be verified. Third, the frequency-band boundaries and the 6:5:1 weighting ratio are dataset-specific modeling choices selected under the adopted validation protocol rather than universal electrochemical constants. Future work should therefore evaluate the framework on larger multi-chemistry datasets, additional cell formats, and EIS measurements obtained under more diverse operating states and temperatures.

## 5. Conclusions

This study proposed a frequency-weighted EIS manifold-learning framework for lithium-ion battery RUL prediction. The main conclusions are as follows:

1. The framework integrates frequency-aware weighting with KPCA-based nonlinear representation learning, followed by RBF–SVR, linear bias correction, and moving-average smoothing to model nonlinear degradation information in EIS spectra.
2. Across seven batteries, the proposed method achieves an average MAE of 6.239 cycles, an average MSE of 59.434 cycles^2^, and an average $R^{2}$ of 0.989, outperforming all evaluated baseline models.
3. Compared with the strongest baseline on each battery, the method reduces MAE by 23.4–33.4% and MSE by 39.0–52.2%. Ablation results further confirm that KPCA provides the primary nonlinear representation capability, while frequency weighting contributes by introducing degradation-related spectral priors before KPCA-based feature construction.

Future work will extend the proposed framework to larger multi-chemistry and multi-format battery datasets, investigate EIS measurements under variable states of charge and more realistic operating conditions, and explore adaptive frequency-weighting strategies to reduce dependence on dataset-specific band settings.

## Figures and Tables

**Table 1:** Consistency analysis of frequency-weighting trends across different battery trajectories.

Battery	Temperature	Weighting Ratio
25C02	25 °C	6:5:1
35C02	35 °C	6:4:1
45C02	45 °C	7:5:1

**Table 2:** Performance comparison of different methods on seven battery datasets. Bold values indicate the best result for each metric and battery.

Dataset	Metric	ANN	GPR	LSTM	SVR	Proposed
	MAE	13.011	8.113	20.713	9.490	**6.216**
25C02	MSE	268.886	84.572	656.274	122.207	**51.625**
	$R^{2}$	0.950	0.984	0.878	0.977	**0.990**
	MAE	13.942	7.700	17.057	8.629	**5.257**
25C05	MSE	324.858	80.011	446.494	103.417	**44.559**
	$R^{2}$	0.940	0.985	0.917	0.981	**0.992**
	MAE	17.883	10.301	20.502	11.560	**7.110**
25C06	MSE	478.556	135.009	560.017	188.860	**72.008**
	$R^{2}$	0.913	0.975	0.898	0.966	**0.987**
	MAE	18.825	11.404	22.003	12.472	**7.747**
25C07	MSE	523.462	165.012	625.023	233.060	**88.828**
	$R^{2}$	0.905	0.970	0.886	0.958	**0.984**
	MAE	15.701	9.300	16.921	10.180	**6.309**
25C08	MSE	360.003	118.031	439.864	153.954	**58.010**
	$R^{2}$	0.932	0.978	0.918	0.971	**0.989**
	MAE	18.183	7.550	9.627	5.965	**4.240**
35C02	MSE	535.531	115.920	208.002	72.625	**35.098**
	$R^{2}$	0.900	0.978	0.961	0.987	**0.994**
	MAE	16.326	10.210	19.101	11.553	**6.794**
45C02	MSE	384.650	138.002	485.010	177.083	**65.909**
	$R^{2}$	0.928	0.974	0.910	0.967	**0.988**

**Table 3:** Model configurations and training settings used in the comparative experiments.

Method	Model Configuration	Key Training Settings
ANN	Standardized raw EIS; MLP with hidden layers (64,32) and ReLU	Adam; lr $=5\times 10^{-4}$; L2 $=10^{-4}$; batch size 32; max. 500 epochs; patience 30.
GPR	Standardized raw EIS; Constant × RBF + White kernel	RBF length scale =1.0; noise level $=10^{-2}$; target normalization; five optimizer restarts.
LSTM	Standardized EIS sequences; two-layer LSTM; hidden size 64; sequence length 5; dropout 0.10	AdamW; lr $=5\times 10^{-4}$; batch size 32; max. 300 epochs; patience 30.
SVR	Standardized raw EIS; RBF kernel	C=100, γ=0.01, ϵ=5; five-fold cross-validation.
Proposed	Frequency-weighted EIS; RBF-KPCA (q=5) followed by RBF-SVR	Weights (6:5:1); $\gamma_{\mathrm{KPCA}}=0.01$; C=100, $\gamma_{\mathrm{SVR}}=0.1$, ϵ=5; bias correction; MA window L=6; five-fold cross-validation.

Note: All data-dependent settings were determined using only the model-development data. Held-out cells were excluded from architecture selection, hyperparameter optimization, and early stopping.

**Table 4:** Component-wise and progressive ablation results on batteries 25C02, 35C02, and 25C07. Bold values denote the best result for each metric and battery. The last two configurations are cumulative additions to weighted KPCA–RBF–SVR.

Configuration	25C02			35C02			25C07		
	MAE	MSE	$R^{2}$	MAE	MSE	$R^{2}$	MAE	MSE	$R^{2}$
Raw EIS + RBF–SVR	9.490	122.207	0.977	5.965	72.625	0.987	12.472	233.060	0.958
PCA + RBF–SVR	10.126	143.684	0.973	5.571	63.842	0.989	13.084	252.840	0.954
KPCA + RBF–SVR	7.731	81.956	0.984	5.143	55.908	0.990	10.411	169.760	0.969
Weighted EIS + RBF–SVR	8.604	101.337	0.981	6.284	82.741	0.985	12.861	241.390	0.956
Weighted EIS + PCA + RBF–SVR	8.917	96.284	0.982	5.862	65.477	0.988	11.706	197.540	0.964
Weighted EIS + KPCA + RBF–SVR	6.784	61.903	0.988	4.572	43.776	0.992	8.612	113.270	0.980
Weighted EIS + KPCA + RBF–SVR + bias correction	**6.083**	56.918	0.989	**4.118**	39.624	0.993	**7.548**	101.870	0.982
Weighted EIS + KPCA + RBF–SVR + bias correction + moving average	6.216	**51.625**	**0.990**	4.240	**35.098**	**0.994**	7.747	**88.828**	**0.984**

## Data Availability

The raw EIS and aging data analyzed in this study were obtained from the publicly available lithium-ion battery degradation dataset reported by Zhang et al. [40]. The processed data supporting the findings of this study are available from the corresponding authors upon reasonable request.
